# Is team science valued in the academic promotions process? A mixed-methods case study

**DOI:** 10.1017/cts.2024.7

**Published:** 2024-01-18

**Authors:** Michael B. Potter, Saji Mansur, Shira P. Rutman, Claire D. Brindis

**Affiliations:** 1 Clinical and Translational Science Institute, University of California, San Francisco, CA, USA; 2 Philip R. Lee Institute for Health Policy Studies, University of California, San Francisco, CA, USA

**Keywords:** Team science, multidisciplinary research, translational research, community engagement, academic promotions

## Abstract

**Introduction:**

Traditionally, research institutions have valued individual achievements such as principal investigator and lead authorship status as primary indicators in the academic promotions process. However, the scientific process increasingly requires collaboration by teams of researchers across multiple disciplines, sometimes including experts outside academia, often referred to as “team science.” We sought to determine whether there is agreement about what constitutes team science at our academic institution and whether current promotion processes sufficiently incentivize faculty participation in team science.

**Methods:**

We conducted 20 qualitative interviews with academic leaders (*N* = 24) at the University of California, San Francisco (UCSF) who supervise faculty promotions processes. Participants were asked to share their definitions of team science and the extent to which faculty receive credit for engaging in these activities during the promotions process. A subset of participants also completed a brief survey in which they ranked the importance of participation in team science relative to other factors that are traditionally valued in the promotions process. Interview data were examined by two analysts using structural coding. Descriptive analyses were conducted of survey responses.

**Results:**

Though team science is valued at UCSF, definitions of team science and the approach to assigning credit for team science in academic promotions processes varied widely. Participants suggested opportunities to bolster support for team science.

**Conclusions:**

Efforts to define and provide transparent faculty incentives for team science should be prioritized at institutions, like UCSF, seeking to advance faculty engagement in collaborative research.

## Introduction

In 2006, the National Institutes of Health (NIH) established the Clinical and Translational Science Awards (CTSA) Program, with the intent of developing infrastructure to catalyze clinical and translational research across multiple disciplines, including collaboration with community stakeholders and policymakers as part of the research process when appropriate. Currently, over 60 medical research institutions across the United States receive CTSA Program funding, and these programs contribute significantly to the advancement of clinical and translational research [1].

Team science is defined by the National Resource Council and the National Academies of Sciences, Engineering and Medicine (NASEM) as “research conducted in an interdependent fashion by more than one individual” [2]. As noted in their related 2015 report, “this simple definition belies the considerable variation within and among science teams,” and terms such as multidisciplinary, interdisciplinary, and transdisciplinary have been used to characterize research teams that integrate knowledge across disciplines. The report deems team science to be central to the clinical and translational research process and calls on academic institutions to provide incentives for their faculty to participate in team science, whether through the restructuring of academic departments, the provision of infrastructure to support team science, or rewards in the context of promotion and tenure review for participation in team science.

The strategic plan put forth by the National Center for the Advancement of Translational Science (NCATS), the NIH agency that administers the CTSA Program, has emphasized the importance of “translational team science” which, “requires a translational research team of scientists, clinicians, research participants, and other stakeholders having a wide range of experience and perspectives” [3,4]. Some experts in this field have made the distinction that “translational teams” are composed of diverse members who interact, adapt, and evolve using established norms and defined roles to address a shared translational objective [5]. Another term supporting this definition and used to describe this type of research is “broadly engaged team science,” which seeks to include key stakeholders in activities across the research spectrum, from generating research questions to implementing research projects, and aiding in the translation of research discoveries to benefit the broader public [6].

Regardless of how team science is defined or encouraged by NCATS and others, the question remains regarding whether incentives to engage in team science are well-aligned with stated values for faculty at academic health centers, especially at early career stages. Traditionally, for career advancement, tenure track faculty must secure principal investigator status on grants and demonstrate first or senior authorship on multiple research publications. Investigators who play other critical roles on research teams, without serving as the lead, may have more difficulty in articulating their contributions and face challenges with career advancement [7,8]. As one commentator wryly asked, “How can academic medical centers avoid stranding their talented faculty in the translational valley of death?” [9].

In response to these calls to incentivize faculty participation in team science, some academic health centers, including UCSF, have begun to affirm the value of participation in team science through the academic review process, as well as evaluation criteria [10,11]. However, although institutions may support team science through documented policies, we have not identified any previous studies that examine whether and how those policies are perceived and implemented by those involved in promotions processes. To better understand the impact of such team science affirmations, we interviewed academic leaders in charge of academic promotions at our institution, asking them to articulate their beliefs about what constitutes team science and how they use it in their deliberations for faculty promotions. Through this formative case study, we aim to begin to develop a roadmap to support the recognition of team science more explicitly and uniformly in our academic promotions processes across our institution and potentially to support similar activities at other academic health centers in the United States facing similar issues.

## Materials and methods

### Study setting

The University of California, San Francisco (UCSF) is an academic health sciences university with schools of medicine, nursing, dentistry, and pharmacy. Together, UCSF’s four schools include 39 academic departments spanning a broad range of basic and clinical sciences. Perennially the top public recipient of NIH research funding, UCSF was awarded $823 million in NIH research funds in 2022. UCSF is also one of the original recipients of CTSA funding, and its Clinical and Translational Science Institute has been continuously funded by NCATS since 2006. At UCSF, the value of “collaborative and team science-oriented research” is affirmed in the faculty handbook, and faculty are invited to highlight these activities on the academic curriculum vitae submitted to departmental promotions committees. Formal criteria for evaluating and crediting such activities in the promotions process are, however, left to department chairs and not institutionally specified.

### Data collection

This mixed-methods study included semi-structured key informant interviews to examine the perspectives of faculty promotions committee members and deans of academic and faculty affairs on team science and collaborative research broadly across the university and specifically, within faculty promotion processes. Interviews included questions about: (1) Definitions of team science; to what extent team science is valued in promotions processes, and whether this differs for faculty in different career tracks and disciplines; (2) Criteria used to evaluate team science participation in promotions decisions; (3) perceptions of institutional support for team science through faculty guidance, mentorship, and resources; and (4) perceptions of differential impact of team science participation for faculty identifying as women or members of minoritized groups. The interview guide is provided as a supplementary file 1.

We also conducted an online 12-question follow-up survey of interviewees as a way of more efficiently capturing interviewee perspectives about the weight given to specific factors considered in the promotions process (see survey elements in Table 1). The complete survey is provided as a supplementary file 2. To avoid duplication or overweighting of responses, only one person per department was invited to complete the survey. Survey respondents were asked to rank a list of factors they use in the promotions process to assess faculty contributions to team science (1 = Not Important to 5 = Very Important) and were provided an open-ended opportunity to share additional measures they may use.

**Table 1.** Interviewees were provided with a supplemental post-interview online survey to assess the relative importance of the following factors in the academic promotions process

Serving as principal investigator of the team
Serving as one of multiple principal investigators on the team
Evaluation of essential contributions by team colleagues
Inclusion of external, non-academic partners on the team
Source of funding brought in by the faculty member to support the team
Amount of funding brought in by the faculty member to support the team
Order in which the faculty member is being listed as author on team publications
Impact of team-based activities on clinical practice guidelines
Impact of team-based activities through widespread dissemination and implementation
Impact of team-based activities on public policies
Impact of team-based activities on local communities

Researchers used purposive sampling to invite academic promotions chairs within 15 departments across the four professional schools within UCSF, including nine in the School of Medicine, and two departments each within the Schools of Dentistry, Nursing, and Pharmacy. Departments were selected to ensure inclusion of both basic and clinical science disciplines. In three departments, promotions leadership activities were shared by more than one person, and for those departments we interviewed two participants. We also interviewed and surveyed key leaders who oversee promotions guidance provided to academic departments and provide final approval of promotions that are recommended: the Associate Dean of Faculty and Academic Affairs for each of UCSF’s four schools and UCSF’s Vice Provost for Academic Affairs.

Interviews and surveys were conducted between July 2022 and October 2022. Interviews averaged 1 hour in length. Prior to the interviews and surveys, the researchers provided participants with an overview of the study purpose, provided assurances of confidentiality, and explained the voluntary nature of participation both in interview invitations via email and at the start of interviews. Interviews were audio-recorded and transcribed. Online follow-up surveys were administered using Qualtrics, a secure online data collection platform [12]. The UCSF Institutional Review Board determined that the project qualified as exempt from oversight (#22-37074).

### Data analysis

Researchers utilized structural coding for qualitative data analysis focused on a list of codes based on the data collection guides and study topics [13]. A subset of transcripts was double coded for inter-coder consistency with an inter-rater reliability kappa level of 0.90. Transcriptions were coded using Dedoose online software version 9 [14]. During the coding process, two researchers discussed code application, definitions, and coding commonalities and discrepancies, with the PI assisting the coders to resolve disagreements until intercoder agreement of greater than 80% was achieved. We did not identify any themes that were not captured within the developed codes. Researchers examined how often themes occurred in the data overall and whether themes emerged in specific types of departments. Survey responses were downloaded into Microsoft Excel for analysis [15]. Researchers conducted descriptive analyses of survey responses.

## Results

We conducted 20 key informant interviews with a total of 24 interviewees, including promotions co-chairs who were invited by promotions chairs in some departments to support the completeness of information provided. All who were invited agreed to participate. Additionally, a total of 19 completed survey responses were received, for a response rate of 95% (one person from each of the 20 interviews was invited to complete the survey on behalf of their department). Interview findings are organized into the following categories that addressed key dimensions of our original research questions: (1) value of team science, (2) differing definitions of team science, (3) processes to assign credit for team science in the advancement process, (4) differences across academic departments, (5) differences across academic career tracks, (6) differences across career stages, (7) impact of team science participation on faculty who identify as women or members of minoritized populations, and (8) promotions committee guidance on the inclusion, description, and evaluation of team science in the academic promotions process. Select survey findings are incorporated within relevant research domain categories where meaningful and relevant. Faculty also provided suggestions for achieving broader consensus and opportunities to strengthen the recognition of team science in the promotions process.

### Value of team science

Nearly all interviewees explicitly acknowledged the potential benefits of a team science approach, which included the quality and richness gained through working across disciplines, and expansion of professional opportunities. One shared, “*I do think that all faculty realize that in order to have impactful studies they need to enlist a variety of different expertise.”* One interviewee spoke about increased access to specialized, expensive, emerging technologies, including computational and biochemical lab genomics approaches, as well as the efficiencies of sharing expertise and resources across teams rather than duplicating efforts within research siloes, “*If you can walk across the quad and tap into somebody’s expertise, that’s going to be more efficient than trying to build that expertise within your own lab.”* However, many interviewees reflected that, while working in multidisciplinary teams has value, it initially takes more time and can slow down the research process when compared to working individually. As one interviewee commented, “*It always takes longer to work as a committee than it does as an individual, but ultimately the work is richer as a group.”*

Several interviewees also described an evolution within academic research with greater recognition of the value of multidisciplinary or transdisciplinary teams as essential to the research process. One interviewee explained this evolution in part as being guided by leadership, “*I think because of the explicit recognition by the more senior members of the faculty that this is (A) the future of science, and (B) a future that we want to embrace – that the nature of science is to be more collaborative going forward.”*

Similarly, there was near unanimity among survey respondents that being lead principal investigator of a team or serving as a multiple principal investigator on a team is “very important” or “important” as a metric in the academic promotions process (*n* = 19). A majority of interviewees and survey respondents indicated that faculty engaged in team science are given credit toward academic advancement if they could demonstrate the impact of their research findings as reflected in widespread dissemination of research findings, changes in clinical guidelines, changes in public policies, or changes in the way the needs of local communities are addressed. However, one survey respondent acknowledged that the full “impact on clinical guidelines, public policies, etc., while important, may be very hard to measure and evaluate for promotions.”

### Definitions of team science

Interviewees were asked to describe how team science-oriented research was defined by their department’s promotions committee and/or by the institution as a whole. Several interviewees were not familiar with the term “team science,” but understood the concept generally as research collaboration, usually across academic disciplines or departments, or different academic institutions, where different members of a team bring unique and essential skills to a project. As one shared, team science involves “*individuals coming together from a variety of disciplines and areas of specialty.*” Some interviewees defined team science as anything involving two or more researchers working together, whether or not they were in different disciplines or departments. Many interviewees reflected on the meaning of team science in terms of skills that are brought to a project, with one sharing, “*It means [each member of the team] bringing something unique to it.*”

Interviewees were asked whether their definition of team science was inclusive of collaboration involving partners from outside of academic settings, such as healthcare or service providers, community representatives, or policy and advocacy group leaders as members of the research team, as incorporated into definitions promoted by NCATS. A few interviewees, mostly in clinical departments with research programs requiring community engagement, acknowledged that community partners can bring important expertise to a research team, with one saying, “*I think there is a greater awareness that this is the future direction, and certainly any time there’s a conversation about equity, I think it becomes very clearly, like, “Who are you listening to and who are you designing around?.”* However, the idea that community partners could be included as members of the research team across the full spectrum of translational research was received with skepticism by many. For example, some interviewees expressed the opinion that collaborators who are not part of academic institutions might provide insights or services, but that they should not be considered part of the research team. One commented about working on research projects with individuals outside of academia, “*Maybe it’s a different kind of a collaboration, but it’s not collaborative science.*”

Qualitative findings were mirrored in survey results. While six of the 19 survey respondents indicated that inclusion of such partners on a research team was “very important” or “important” in assessing academic promotions, six others reported this as only “somewhat important” and seven reported this as “less important” or “not important.”

### Processes to assign credit for team science

Interviewees were asked about the ways in which team science is credited in faculty advancement decisions. Several interviewees explained that the philosophical value of team science within the university was not manifesting in decision-making for academic promotions at a department level. As one shared:

*The goals are there, the process allows for it, but I don’t think it’s being actuated in a way that success in team science is being recognized, as opposed to the number of publications, the number of committees that somebody is sitting on, the teaching that they’re doing, and the other big categories that fit into the resume.*

Many interviewees shared that the traditional metrics for assigning credit to faculty continue to be primary in the advancement process. These include independence as assessed through a principal investigator role on research grants and authorship order in peer-reviewed publications (first or senior). One interviewee noted, *“The bottom line is collaborative research is nice, it’s important, and it helps all of us,”* but went on to explain that traditional metrics of individual achievement are a prerequisite for advancement. Yet, a couple of interviewees described a perceived shift with collaborative papers and co-senior authorship becoming more common, with one from a basic science focused department sharing, “*The department, I think, now evaluates collaborative papers equivalently to kind of stand-alone papers where there is one senior author with no shared credit, at least in the author list.”*

The responses to the surveys confirmed this shift, in that only seven respondents reported first or senior authorship as “very important” or “important” to the promotions process, while 14 survey respondents reported that formal evaluation of the individual’s essential contributions to the research team was “very important” or “important.” However, there were questions about the best way to accomplish this type of assessment. In open-ended survey responses, one stated that the “*impact on the community is harder to measure – I’d love to know what metrics are being used by others.*” One suggested that the best way to do a formal evaluation would be through a “*faculty summary of their own work and impact, solicited letters, and products disseminated*.” Another suggested that “*invited presentations, keynote addresses and participation on scientific advisory boards*” could be metrics of successful team science impact.

### Differences across academic departments and disciplines

Interviewees were asked whether collaborative and team science research is valued in the promotions process differently for faculty within different types of academic departments (e.g., those emphasizing clinical or bench science). The overall sense was that such differences do exist. One interviewee described that, *“The conceptual perspective and value that different division heads put on the kind of research we’re talking about here differs.”* This was seen in that promotions chairs from larger and more clinically focused departments generally described a greater value being placed on team science than those representing smaller and more basic science-oriented departments. One interviewee from a larger clinically focused department shared, *“It’s an expectation that they be involved in team science… [it’s] a big part of the review.”* One interviewee from a basic science department perceived an evolution in how basic science departments are beginning to appreciate the importance of team science, stating that, *“Now biology is shifting towards increasing collaboration as a way of doing science.”* However, this general statement did not assign value to such collaboration in the academic promotions process.

### Differences across academic career tracks

Interviewees were asked whether they perceived team science as being valued differently within different academic career tracks (e.g., tenure track researchers, adjunct faculty, clinician scholars, or clinical health care providers). Most interviewees shared that there is an expectation that tenure track researchers must establish an independent line of academic research to be promoted. One interviewee specified that faculty in this career track, “*should have independent funding, extramural funding, as a [principal investigator] ideally, as you get to associate and full professor.*” Interviewees described that faculty in other academic career tracks could be successful in the promotions process with a broader academic portfolio that highlights team science achievements. As one interviewee explained, those in more clinical tracks are, “*more likely to participate in team related science*” and to get credit for it in the academic promotions process, even when their contributions to those teams may be more on the level of facilitation or collaboration with less clearly defined scientific contributions.

### Differences across career stages

Several interviewees explained that research faculty who focus most of their academic activities on being a part of larger multidisciplinary research teams are at a disadvantage in the promotions process, especially in the early stages of their careers, as one interviewee stated:

*At the first stage, you feel like you have to differentiate yourself as a person, as a researcher, as somebody who’s successful in their own right. And it feels like you are disadvantaged if what you’re leading with is a portfolio of work that is team science, because our evaluation criteria and process and tradition is about individual success.*

According to two interviewees, junior research faculty may also be discouraged from participating on research teams that include their mentors, with one noting:

*Publishing with prior mentors is still discouraged. So that is a subclass of collaboration that I think is still discouraged within the department – the idea being that once you leave your post doc, you really should be establishing something new and not something derivative of your prior training. And so, if you continue to publish with that prior mentor, it becomes unclear what that new thing that you’re doing actually is.*

Summarizing the importance of considering differences among faculty stages and career paths, one survey respondent wrote that the weight assigned to various dimensions of team science should *“vary depending on the series, rank, and other factors, such as field of study or expertise. A holistic approach is [or should be] used for academic review.”*

### Impact of team science participation on the promotions process for faculty who identify as women or members of minoritized populations

Interviewees were asked to consider how the assessment of team science in the academic promotions process might uniquely affect faculty who identify as women and/or members of minoritized populations. There was a perception among several interviewees that women faculty, particularly at earlier career stages, may experience challenges to participating in team science given the time it takes to develop collaborative and team science-oriented research balanced with the time pressures of maternity leave and childcare responsibilities. Some interviewees perceived that faculty from minoritized groups might actually be more likely than others to want to participate in team science, especially in team-based research that broadly engages with communities that they represent. However, the extra burden of departmental service that is often experienced by minoritized faculty groups may lead to less opportunity to participate in time-intensive forms of team science [16]. In contrast, one interviewee stated that participation in team science by faculty from underrepresented groups provides opportunities for networking and “*a sense of belonging and connectedness,*” which help buffer experiences of structural racism and support “*a stronger portfolio.”*

Asked whether the undervaluing of team science in the promotions process might discourage women and minoritized faculty from seeking tenure or staying at the university, one interviewee stated, “*We are very attentive to implicit bias and we always bring up those issues at the table. So, I don’t think there’s a pattern.”* However, most interviewees indicated that they had either not considered this issue or that they were not certain whether it was a problem that needed to be addressed because in actuality there were relatively smaller percentages of these groups represented in their faculty.

Survey responses reflected a consensus about the value of recognizing and promoting those whose team science research is serving the direct needs and priorities of represented communities, including those that have been historically marginalized or oppressed. Twelve survey respondents indicated that conducting and rewarding this type of team science research is either “very important” or “’important,” and five more indicated that it is “somewhat important” as part of the promotions process.

### Promotions committee guidance on the presentation and evaluation of team science in the academic promotions process

The value of “collaborative and team science-oriented research” is affirmed in the UCSF faculty handbook, and faculty are invited to highlight these activities on the academic curriculum vitae that is submitted to departmental promotions committees. Formal criteria for defining and evaluating such activities are, however, left to department chairs and not institutionally specified. In spite of written policy supporting team science, interviewees in charge of academic promotions processes had varying levels of awareness about specific instructions to faculty for highlighting contributions to team science in the academic promotions process. Several interviewees noted that faculty often fail to fully enumerate their team science activities in promotions application materials. This was in-line with comments by many interviewees who described a lack of consistent or explicit guidance to faculty members who are up for promotion, or to promotions committee members who are involved in the evaluation process. As one academic promotion chair shared, “*The executive committee picks the person that we think is best qualified to review that person’s science… we don’t actually tell them anything specifically about how to judge it.”*

## Discussion

As major national governmental and non-governmental research-focused institutions such as NCATS and NASEM increasingly call for a greater recognition of the value of team science, it is important to assess whether institutional incentives, as reflected in academic promotions processes, are sufficiently in place to support faculty participation in team science across the research enterprise. In this study, we document both the inroads and the barriers that exist at UCSF, which is recognized for its success in securing NIH funding and in the scientific achievement of its faculty across the spectrum of clinical and translational research.

First, there appears to be a growing recognition of the value of team science. In part, this reflects pragmatic considerations, such as efficient use and sharing of technical expertise and expensive resources. Additionally, there is momentum toward recognizing that many of the most important research questions facing society cannot be effectively addressed without harnessing the power of diverse disciplines, methodologies, and perspectives. During the course of our interviews, we learned of activities in certain departments to assure that junior faculty receive mentoring to assure them that participation in team science will not be an impediment to academic advancement at UCSF. One department includes the statement that, in the promotion to associate professor, “*collaborative research accomplishments may be considered if it is clearly documented that the faculty member has made essential, unique, and independent contributions to the work*.” Another department provides the following guidance, *“Although the university and the department value traditional “independent investigator” metrics such as serving as PI on grant awards and as first or senior author on peer-reviewed publications, this section should also comment where applicable on the faculty member’s role in collaborative research projects and contributions to team science.”* However, for the vast majority of departments, faculty receive little or no formal guidance on what information regarding team science activities should be described as part of the promotion packet or even where to formally note such activity. We also did not learn of any department that provides metrics for team science achievements, which may lead to confusion among faculty who are engaged in the academic promotions process, as well as among the individuals who are responsible for reviewing their promotions packets.

At the same time, we frequently heard that UCSF faculty know what they must do to be promoted, and that few if any faculty who excel at team science are held back due to lack of evidence of individual achievement. In addition, several interviewees indicated that, while team science achievement is “nice to have,” it was not as important as more traditional metrics like being a principal investigator on a federally funded grant. We observed that some respondents endorse a false dichotomy, where demonstrating research leadership, independence, and scholarly contributions is not possible for those who invest too much in the tenets of team science. As a result, junior research faculty with much to contribute to such activities may view participation in team science as a time-consuming risk that they cannot afford to take.

Simultaneously with efforts to encourage the development of multidisciplinary and transdisciplinary research teams, there is increasing recognition that, in many cases, community partners and/or non-academic institutions can and should be essential contributors or members of the research team. For example, community-based expertise may be needed to generate and prioritize research questions, to build trusting relationships with communities affected by the research, to identify best practices for participant recruitment across diverse communities, to develop strategies for study implementation, and to assist with dissemination of findings in ways that will assure optimal impact of the research findings [6]. From this perspective, research that fails to include community partners will at best miss critical opportunities and at worst create more harm than benefit for the communities affected by the research. However, developing broadly engaged team science research programs takes time, and research faculty who fail to be granted the time and resources to invest in authentic relationship building efforts may be penalized in the promotions process or turn their attentions to other academic activities instead. The lack of resources may furthermore discourage promising trainees and faculty, who would like to engage in team science with such communities, from seeking faculty research positions in the first place.

Faculty from underrepresented and minoritized groups within academic institutions, by virtue of their lived experiences, are often ideally situated to be leaders and/or key contributors to broadly engaged research teams. Academic institutions that fail to value community-engaged team science may find themselves at a disadvantage in the recruitment, retention, and promotion of these talented individuals. Though UCSF is a national leader in its efforts to promote a diverse workforce [17], there is still much to be done. In this study, we found that some academic departments may not yet recognize the potential importance of supporting broadly engaged team science as part of these efforts.

Finally, we learned through this study that departmental promotions chairs and academic deans continue to struggle with definitions of team science and how it should be recognized and credited for an extremely heterogenous group of faculty whose work spans the full range of discovery across the spectrum of bench, clinical, translational, and population sciences and which also includes many faculty, such as clinicians and educators, whose job descriptions do not include the expectation to participate in research. Rather than a single definition and approach to the evaluation of team science in the promotions process that applies to all, it may be more useful for departments to examine the definitions, expectations, and incentives for participating in team science, perhaps with tailored approaches for faculty in different career tracks. We believe that these definitions and guidelines should be developed by diverse stakeholders in the academic promotions process, endorsed at the level of academic deans and academic promotions chairs, and shared with members of academic promotions committees. Clarity across the continuum of all stakeholders, from faculty who are responsible for conducting promotion application reviews within the department, as well as the campus review, to the mentors who work with faculty as they are recruited and hired, to the faculty members themselves who are charting their careers, will all help assure greater consistency.

Shared definitions and concrete examples of team science may also help provide the framing and range of what constitutes scientific rigor, merit, and scientific contribution. Furthermore, within the criteria of what constitutes team science, building in sufficient time flexibility that acknowledges and takes into account that some types of team science may take longer to conduct and generate the types of scientific results or products that are traditionally recognized in the promotion process, may also be necessary. Greater consensus about the pathways toward achieving excellence in team science may be achieved by the development of trainings for junior faculty and their mentors, supported by new and evolving models such as those recently published by Brasier, et al, and by formal adoption or endorsement of resources developed by NIH [18–21]. Junior faculty who engage in team science may require guidance on ways to ensure the team has well-defined roles that can be described and evaluated, and that their unique contributions can be clearly recognized in ways that enhance their career development. Metrics to assess the impact of such initiatives on participation in team science will be needed, with special attention to faculty recruitment, retention, and equity in promotions of faculty who identify as women and/or who are members of minoritized populations. In Table 2, we present key recommendations from this study that have been presented to our institutional leaders and which may serve to inform the activities of others seeking to expand incentives for participation in team science at their institutions, as well.

**Table 2.** Recommendations to promote team science in academic promotions

Definitional clarity	Define excellence in Team Science, specific to different career stages, career tracks, and disciplines
	Incorporate expertise from community-based partners and partners outside of academic institutions in definitions of Team Science (i.e., Broadly Engaged Team Science)
	Embed Team Science definitions more prominently into instructions for faculty about preparing their academic promotions materials
Promotions institutional systems and structures	Provide guidance and training for departmental promotions chairs and committee members on valuing and assessing Team Science in academic promotions materials, as an alternative metric or in addition to other more traditional metrics of academic achievement
	Continue and bolster support for Team Science through intramural funding opportunities and interdisciplinary research programs, including seed grants open to all faculty, and departmental budgets to incentivize Team Science engagement
Training, mentoring, and support of faculty	Provide trainings and workshops for junior faculty and their mentors on Team Science definitions and how to represent Team Science excellence in academic promotions materials
	Explore opportunities to recognize exemplary teams, instead of individual researchers, through university-wide chancellor’s awards for groundbreaking Team Science collaborations

In summary, our study indicates that our institution has made strides toward the encouragement and recognition of team science through its academic promotions process, but culture change can be a slow process, and there is more to be done. We hope to use the study findings to further catalyze changes in the evaluation and recognition of team science in the promotions process at our institution and offer our findings other institutions, which may have similar experiences and goals.

## Supporting information

Potter et al. supplementary material 1 Potter et al. supplementary material

Potter et al. supplementary material 2 Potter et al. supplementary material

## Supplementary material

The supplementary material for this article can be found at https://doi.org/10.1017/cts.2024.7.
