# Supplementary material for: Is team science valued in the academic promotions process? A mixed-methods case study
Source: J Clin Transl Sci. 2024 Jan 18;8(1):e28. doi: 10.1017/cts.2024.7 (PMC10880000; doi:10.1017/cts.2024.7)
Supplement: Potter et al. supplementary material 1 — Potter et al. supplementary material [file S2059866124000074sup001.docx]

Team Science and Academic Promotions Interview Guide

Briefly, please tell us your role(s) in the Academic Promotions process in your department.

How long have you been in this role?

First, we would like to share with you the current UCSF instructions for faculty members within the Academic Promotions portal:

*A description of your current research interests/program is required for Ladder Rank, In Residence, Clinical X, and Adjunct faculty. Health Sciences Clinical faculty should include this description as appropriate. Collaborative and team science-oriented research is valued at UCSF through the academic review process. Highlight important contributions to collaborative research (i.e., unique, essential, and creative contributions), particularly those that have led to significant grants and papers for which you were not necessarily PI or first/last author, especially if they are important to your identity as a researcher.*

***Now, we would like to ask you some questions.***

***Definitions and Involved Partners***

1. To respond to the instructions we shared, can you please describe how “collaborative and team science-oriented research” is defined by your department’s promotions committee?
2. When you think of team science, what kind of collaborative partners come to mind first? (For example, what roles are they in? What institutions, departments, or organizations are they based in?)

Possible probe, if not mentioned: More specifically, is your definition of team science inclusive of collaboration involving any of the following?

- 1. Across UCSF departments (e.g., inclusive from across basic science, social science, and/or clinically-oriented departments)
  2. Health-focused research faculty at other academic institutions (which may be in the same or different academic disciplines)
  3. Researchers in disciplines outside health care (e.g., economists, engineers, etc.)
  4. Patients or patient advisory groups
  5. Community-based partners (e.g., healthcare providers, service providers, researchers, community representatives, etc.)
  6. Policy/advocacy group leaders

1. How is team science valued by your department’s promotions committee? Are some types of team science collaborations more highly valued by your department than others? Please elaborate.
2. Can you please discuss specifically whether your department’s promotions committee defines community partnerships as a part of collaborative and team science-oriented research? Do you view it as separate and why/why not?
3. Is collaborative and team science-oriented research valued in the same way in the promotions process for all faculty in your department? If not, can you share where you have seen differences?

Possible probe:

- 1. How, if at all, is team science valued differently in the promotions process for different academic series (Ladder Rank, In Residence, Clinical X, HS Clinical, Adjunct)?
  2. Is team science valued differently in the promotions process for different ranks (Assistant/Associate/Full professor)?
  3. How about for population health vs. clinical trials vs. bench research?

1. Do you provide guidance to your promotions committee members about how they should evaluate the quality of collaborative and team science-oriented research in this section of their academic curricula vitae? If yes, what evaluation criteria are used? If you have a written policy or guidance document on how promotions committee members should evaluate this portion of the advance application, can you share it with us?
2. Do you believe that faculty receive consistent or inconsistent advice and/or guidance regarding the value of collaborative and team science-oriented research and how to describe it work in this area as part of their academic review?

For example, is guidance from your department the same as what is provided by division-based peers/mentors/leaders, and campus external groups, such as the Vice Provost for Academic Affairs/Committee on Academic Personnel/Dean’s Office?

***Faculty Development***

1. Do you believe that your faculty members are encouraged to engage in collaborative and team science-oriented research during the recruitment process? What about during the onboarding process? Can you share examples of this?
2. Do your faculty members have access to training on how to conduct collaborative and team science-oriented research? Do you think such training would be valuable? If so, what types of skills would be important to include in the training?
3. Are faculty offered resources by your department (e.g., protected time, funding, mentorship) to build competency in collaborative and team science-oriented research?
4. In what specific ways do your department’s mentors and sponsors support collaborative team science, for example, by building team science into individual development plans, or other efforts?
5. Are there steps the department or campus could take to further promote/support faculty to pursue collaborative and team-science research?

***Process***

1. Do you believe that allowing your faculty to describe their collaborative and team science-oriented research in their academic curricula vitae has made a difference in the academic promotions process in your department? If so, how? Please provide two relevant examples.
2. Do you believe this section of the academic curricula vitae should be altered or enhanced? If so, how?
3. Are there steps the department could take to increase transparency/communication about the value of collaborative and team science-oriented research? Please explain.

***Career Impact***

1. Do you believe that UCSF’s inclusion of collaborative and team science-oriented research as a consideration in promotions is helpful or harmful to faculty promotion in your department? Please explain.
2. What, if any, other challenges do faculty who focus on collaborative and team science-oriented research face in your departmental promotions process? Please explain or provide specific examples.
3. Can you please talk about the impact on promotions specifically of female faculty who focus on collaborative and team science-oriented research? What about faculty from under-represented groups? Please explain.

***Summation***

1. Have you observed changes in how your department approaches the evaluation of collaborative and team science-oriented research in the promotions process over time?
2. Is there anything else that you would like to add?

Thank you!
