# Supplementary material for: Is team science valued in the academic promotions process? A mixed-methods case study
Source: J Clin Transl Sci. 2024 Jan 18;8(1):e28. doi: 10.1017/cts.2024.7 (PMC10880000; doi:10.1017/cts.2024.7)
Supplement: Potter et al. supplementary material 2 — Potter et al. supplementary material [file S2059866124000074sup002.docx]

Team Science and Academic Promotions Post-Interview Survey

Please rank, based on your experience, how important each factor listed is in assessing the value of a faculty member’s contribution to collaborative and team science-oriented research in the promotions process:

|  | Not Important  1 | Less Important  2 | Somewhat Important  3 | Important  4 | Very  Important  5 |
| --- | --- | --- | --- | --- | --- |
| Serving as the lead PI of the team |  |  |  |  |  |
| Serving as multiple PI |  |  |  |  |  |
| Formal evaluation of the individual’s essential contributions to the team by their team colleagues |  |  |  |  |  |
| Inclusion of external, non-academic partners on the team (e.g., patient representatives, community-based partners, policy advocates, etc.) |  |  |  |  |  |
| Source/Type of funding that is brought in by the faculty member to support the team (e.g., NIH vs. other) |  |  |  |  |  |
| Amount of funding and overhead brought into your department by the team |  |  |  |  |  |
| First or senior authorship on research team publications |  |  |  |  |  |
| Impact on clinical practice guidelines resulting from team-based activities |  |  |  |  |  |
| Impact on public policies resulting from team-based activities |  |  |  |  |  |
| Impact as reflected in widespread dissemination and implementation of research findings from team-based activities |  |  |  |  |  |
| Impact on the needs of local communities (including those that have been historically marginalized and/or oppressed) resulting from team-based activities |  |  |  |  |  |

Are there other metrics you use in assessing the value and importance faculty involvement in collaborative and team science-oriented research in the promotions process? If yes, please share them below:

Thank you!
